# Effects of Chicory on Serum Uric Acid, Renal Function, and GLUT9 Expression in Hyperuricaemic Rats with Renal Injury and* In Vitro* Verification with Cells

**DOI:** 10.1155/2018/1764212

**Published:** 2018-12-02

**Authors:** Yong-Nan Jin, Zhi-Jian Lin, Bing Zhang, Yun-Fei Bai

**Affiliations:** ^1^Department of Clinical Chinese Pharmacy, School of Chinese Pharmacy, Beijing University of Chinese Medicine, Beijing 100029, China; ^2^Department of Integrated TCM and Western Medicine, Yanbian University Hospital, Yanji 133000, China

## Abstract

Hyperuricaemia (HUA) is an independent risk factor for chronic kidney disease. Urate crystals are deposited in the kidney and can cause renal tubular interstitial fibrosis, leading to renal dysfunction. Chicory extract (hereafter referred to as chicory) clearly reduced serum uric acid levels in rats with HUA induced by 10% fructose. This is the first study to observe the effect of chicory on serum uric acid levels and renal function in rats with HUA and renal injury. In vivo studies using hyperuricaemic rats with renal injury induced by yeast and adenine demonstrated that chicory decreased serum uric acid level, and its effect of delaying the progression of kidney injury was better than that of benzbromarone. In vitro cell experiments showed that this effect is related to the inhibition of GLUT9 protein expression in renal tubules and that lowering blood uric acid concentrations is one of the factors that alleviates renal damage. The results of this study indicate that chicory can be used as an alternative for alleviating renal dysfunction in hyperuricaemia.

## 1. Introduction

Hyperuricaemia (HUA) is a metabolic disorder associated with an abnormally high uric acid level in blood, and HUA is an independent risk factor for chronic kidney disease [1, 2]. As reported in the literature, the risk of developing kidney disease and the risk of worsening renal function increases by 71% and 14%, respectively, for every 1 mg/dl increase in serum uric acid, and the risk of developing new kidney disease is 3 times higher when the serum uric acid level exceeds 9 mg/dl [3].

Approximately, ninety percent of hyperuricaemia cases are caused by insufficient excretion of uric acid [4], and uric acid homeostasis in the body is controlled by the synergistic effect of a number of urate transporters in kidney and intestine. The urate transporters are divided into urate reabsorption transporters and urate excretion transporters. The former includes URAT1 (SLC22A12), OAT4 (SLC22A11), OAT10 (SLC22A13), GLUT9 (SLC2A9), and the latter includes OAT1 (SLC22A6), OAT3 (SLC22A8), MRP2 (ABCC2), MRP4 (ABCC4), NPT1 (SLC17A1), NPT4 (SLC17A3), UAT, and ABCG2 [5–8]. In the kidney, as the urate transporters decrease the reabsorption of uric acid and increase its secretion, they may provide new strategies for the treatment of hyperuricaemia related diseases such as gout, hypertension, diabetes mellitus, coronary heart disease, and chronic kidney disease.

Antihyperuricaemia drugs inhibit or activate these transporters, thereby increasing uric acid excretion in the kidneys to lower blood uric acid levels. Although their pharmacological effect of reducing serum uric acid is clear, these drugs do not effectively delay the progression of renal injury caused by high blood uric acid [9].

*Cichorium intybus* L., also known as chicory, is a perennial herb belonging to the Asteraceae family. Chicory is regarded as a diuretic and choleretic in Uighur folk medicine and is commonly used in the treatment of jaundice, edema and oliguria [10]. Chicory has some pharmacological effects, such as anti-diabetic, anti-inflammatory, antioxidant, and antihyperlipidaemic effects [11].

In a previous experiment, we found that chicory could decrease serum uric acid levels by inhibiting the activity of xanthine oxidase (XOD), the enzyme that catalyses the generation of uric acid from hypoxanthine and xanthine, and by promoting uric acid excretion by upregulating the mRNA expression of OAT3 in hyperuricaemic rats [12, 13]. However, the effect of chicory on renal uric acid excretion in hyperuricaemic rats with renal injury is unknown, and no studies have been conducted on the effects of chicory on the above mentioned indicators in hyperuricaemia with renal injury. In addition, it is considered that kidney is responsible for the excretion of 66% of uric acid, and GLUT9 plays an important role in as a high capacity urate transporter in renal uric acid excretion.

Therefore, the objective of this study was to examine the effect of chicory on serum uric acid and renal function in hyperuricaemic rats with renal injury through renal urate excretion and GLUT9 expression based on our previous research. In vitro cell experiments were used to verify the effect of GLUT9 expression on uric acid transport.

## 2. Materials and Methods

### 2.1. Drugs

Yeast was purchased from OXOID (UK) and dissolved in purified water (1 g:1 ml) before being given rats. Adenine was purchased from Sigma-Aldrich (USA) and suspended in purified water (80 mg:1 ml) before being given rats. Benzbromarone tablets were purchased from Heumann Pharma GmbH (Germany). The chicory used in the study was authenticated by Professor Yan (Traditional Chinese Medicine Appraisal Teaching and Research Section of Beijing University of Chinese Medicine). Chicory was crushed and ground into powder. The powder was extracted with distilled water (1 g:10 ml) by heating to reflux for 1 h twice. The solution was concentrated by a rotary evaporator after filtering and diluted to different volumes with purified water [13].

### 2.2. Animals

Sixty male Sprague-Dawley (SD) rats (240-260 g body weight) were purchased from Beijing SPF Laboratory Animal Technology Co., Ltd. (Certificate of Quality: SCXK-2016-0002). The animals were housed in an air-conditioned room where the temperature and humidity were 20-24°C and 45-55%, respectively, with 12 h light-dark cycles. Food and water were provided ad libitum. After 5 days of acclimation, the rats were randomly divided into 5 groups (n=12): control group (CG), hyperuricaemia with renal injury group (MG), benzbromarone group (BEN), high dosage of chicory group (HD-C), and low dosage of chicory group (LD-C). Normal rats were used for the CG, and hyperuricaemia with renal injury was induced in the rats of the other groups by intragastric administration of yeast (15 g·kg^−1^·d^−1^) and adenine (80 mg·kg^−1^·d^−1^). After 8 h, the treatment groups were intragastrically administered benzbromarone (20 mg·kg^−1^·d^−1^) or chicory at a high dosage (13.2 g·kg^−1^·d^−1^) or low dosage (6.6 g·kg^−1^·d^−1^), and the CG and MG rats were intragastrically administered an equal volume purified water at the same time. The animal experiments lasted 5 weeks. The animal study protocol was approved by the Animal Care and Ethics Committee of Beijing University of Chinese Medicine.

### 2.3. Sample Collection and Index Detection

Blood samples were collected from the tail tips after 12 h of fasting at weeks 1, 3, and 5. The serum was separated and stored at -20°C. The 24-h urine of ten rats per group was collected by the metabolic cage method at weeks 1, 3 and 5. The urine volume was recorded, and then the supernatant was taken and stored at -20°C. Serum uric acid (SUA) and serum creatinine (SCr) levels were measured according to the instructions of a uric acid assay kit (BioSino, China) and CRE assay kit (Nanjing Jiancheng, China), respectively. Additionally, urinary uric acid (UUA) and urinary creatinine (UCr) were measured according to instructions of the same kits. Urinary microalbumin (UMA) was measured according to the microalbumin assay kit (Nanjing Jiancheng, China). The 24-h UUA, 24-h urinary microalbumin and creatinine clearance (CrCl) were calculated by the following equation:

1. 24-h UUA (mg/d) = [UUA (*μ*mol/L)]×[24-h urine volume (ml/d)]×168.11 g/mol×10^−6^;
2. 24-h UMA (mg/d) = [UMA (mg/L)]×[24-h urine volume (ml/d)]×10^−3^;
3. CrCl (ml/min) = [UCr (*μ*mol/L)]/[SCr (*μ*mol/L)]×[urine volume per minute (ml/min)].

After 5 weeks of treatment, all animals were anaesthetized with 10% chloral hydrate solution (3.5 ml/kg), and blood samples were collected from the abdominal aorta after 12 h of fasting. The blood samples were analysed as described above. The upper half of the left kidney was fixed with 4% paraformaldehyde for histological evaluation, and the lower half was preserved at -80°C for western blot and qPCR assays.

### 2.4. Renal Histological Evaluation

The left kidneys were fixed with 4% buffered paraformaldehyde, dehydrated with 50-100% ethanol and embedded in paraffin. Samples were cut into 3 *μ*m sections and stained with haematoxylin-eosin (HE) for histological observation. The observation and capture were performed with an automated upright microscope system (Olympus BX53, Japan).

### 2.5. Determination of the Effect of Chicory on GLUT9 mRNA Using qPCR

Total RNA from the kidneys was extracted by Trizol reagent (Thermo Scientific, USA). The concentration of total RNA was measured with a UV-Vis Spectrophotometer Q5000 (Quawell, USA), and the integrity was evaluated by electrophoresis in a 1% agarose gel. The RNA was reverse-transcribed following the manufacturer's protocol of the Revert Aid First Strand cDNA Synthesis Kit (Thermo Scientific, USA). Quantitative real-time PCR was performed using iTaq™ Universal SYBR Green Supermix (Bio-Rad, USA) and CFX96™ (Bio-Rad, USA). The forward and reverse primers for GLUT9/SLC2A9 were designed according to the mRNA sequences shown in Table 1 (GenBank NCBI Reference Sequence: NM 001191551.1). The forward and reverse primers for GAPDH were from a literature report [11]. Amplification of PCR fragments spanning different exons was used to prevent contamination by genomic DNA. The final reaction volume of each sample was 20 *μ*l. The relative mRNA expression levels of the target genes in each sample were calculated using the comparative CT method. The GLUT9 mRNA expression values were normalized to the house keeping gene GAPDH to determine the relative expression ratios for each mRNA relative to the CG.

### 2.6. Determination of the Effect of Chicory on GLUT9 Protein Using Western Blotting

The proteins were extracted by lysing the kidney tissue with RIPA (Solarbio, China). The concentration of the proteins was measured by a BCA protein assay kit (Solarbio, China). The proteins were mixed with 4×SDS buffer (Solarbio, China), heated for 10 min and then separated on 10% SDS-PAGE gels. Proteins were transferred to PVDF membranes (Millipore, USA), and the membranes were blocked with TBST (containing 5% skim milk) at room temperature for 1 h. Then, the membranes were incubated with rabbit anti-GLUT9 antibody (1:2500, Millipore, USA) and rabbit anti-*β*-actin antibody (1:5000, Proteintech, USA) at 4°C overnight. The next day, the immunoreactive bands were detected using goat anti-rabbit IgG H&L (1:10000, Abcam, USA) and goat anti-mouse IgG H&L (1:10000, Proteintech, USA) as the secondary antibody at room temperature for 1 h. The protein blots were visualized using ECL immunoblot detection reagent (Millipore, USA). The density of bands was analysed by Image J and normalized to *β*-actin. Protein expression was quantified as the ratio of the specific band to *β*-actin.

### 2.7. Cell Experiment

#### 2.7.1. Cell Culture

The HKC cell line was purchased from the National Infrastructure of Cell Line Resource & Peking Union Medical College Cell Resource Center. The HKC cells were grown in complete medium consisting of DMEM/F12 (Corning, USA) with 5% fetal bovine serum (FBS;* Sijiqing*, China), 1% nonessential amino acid (NEAA; Corning, USA), and 1% penicillin streptomycin solution (Corning, USA) and cultured at 37°C under 5% CO_2_-95% O_2_ conditions. The cells adhered to the wall and grew for 4-5 days for one generation.

#### 2.7.2. Determination of the Proliferation of HKC Cells Using the MTT Method

HKC cells (1.0 × 10^4^ cells/ml) in the logarithmic growth phase were seeded in 96-well plates (200 *μ*l/well). PBS was added to the peripheral wells (200 *μ*l/well), and the HKC cells were cultured for 24 h. Then, the culture medium was replaced with new culture medium (200 *μ*l/well) containing various concentrations of uric acid (UA, Sigma-Aldrich, USA), BEN (China Pharmaceutical Biological Products Verification Institute, China), and chicory (Chi, BUCM, China) (n=6). After 24 h and 48 h, MTT solution (5 mg/ml) was added (20 *μ*l/well). Following incubation at 37°C for 4 h, the supernatant was gently removed, and DMSO was added (150 *μ*l/well) to dissolve the formazan crystals completely by oscillating for 10 min on a rotator under dark conditions. Finally, the absorbance of each well was measured at 490 nm wavelength using the Enzyme Microplate Reader (Thermo Scientific, USA).

#### 2.7.3. Determination of the Effect of Chicory on the HKC Cells GLUT9 Protein Using Western Blotting

The HKC cells were incubated in complete medium containing UA, BEN, and Chi for 24 h, and then the cells were collected and lysed by RIPA (Solarbio, China). The total protein was extracted and denatured by heating in a water bath for 10 min. The proteins (5 *μ*g) were separated by 10% SDS-PAGE under a constant current of 300 mA for 90 min. The next procedures, including blocking with milk, primary and secondary antibody incubation, protein blot visualization, and quantification, were the same as described in Section 2.6.

#### 2.7.4. Determination of the Effect of Chicory on the Transport Ability of GLUT9 Using the Transwell Assay and HPLC

HKC cells (1.0 × 10^5^ cells/ml) in the logarithmic growth phase were added to the upper chamber (500 *μ*l/well) of 12-well Transwell plates (0.4 *μ*m, Corning, USA), and complete medium was added to the lower chamber (1.5 ml/well). The HKC cells were cultured on Transwell polycarbonate membranes for 12 days. The transepithelial electrical resistance (TEER) of the cell monolayer was measured by an Epithelial Volt-Ohm Meter (Millicell ERS-2, Millipore, USA), and the culture medium was replaced every 2 days. The UA transport experiment began when the TEER gradually increased and tended to be stabilized.

First, the culture medium in both the upper and lower chambers was removed, and the HKC cell monolayer was washed twice with 37°C HBSS. Complete medium (1.5 ml) containing the specified concentrations of BEN, Chi, and UA was added to the lower chambers, and the complete medium of the CG contained UA only. Then, 0.5 ml of HBSS was added to the upper chambers. After 30, 60, 90, and 120 min, 300 *μ*l of liquid was taken from the upper chambers, and 300 *μ*l of HBSS was added every time to ensure that the volume of HBSS in the upper chamber was 0.5 ml. The total amount of UA transport at each time period is calculated from the following formula:

(1) $$\begin{array}{c} \Delta Q=C_{n}•V_{AP}+C_{i}•V \end{array}$$

*ΔQ* is the total amount of UA transport at each time period;* C*_*n*_ is the concentration of UA in the liquid collected at the previous time point; *V*_*AP*_ is the upper chamber volume (0.5 ml);* C*_*i*_ is the concentration of UA in the liquid collected at each time point;* V* is the volume of liquid collected from the upper chamber every time (0.3 ml).

The liquid samples were filtered through a 0.45 *μ*m filter, and the UA content in each sample was determined using an Agilent 1100 Series HPLC system (Agilent Technologies, USA) equipped with a photodiode array detector. An Agilent Eclipse XDB-C_18_ (5 *μ*m, 250 mm × 4.6 mm, Agilent, USA) column was used with a mobile phase consisting of solvents A (6%; methanol) and B (94%; water containing 0.2% acetic acid); the flow rate was 1.0 ml/min. The column temperature was maintained at 30°C, and the detection wavelength was set at 288 nm. The sample volume was 20 *μ*l.

### 2.8. Statistical Analysis

Statistical analyses were conducted by SPSS 20.0 software. All data were expressed as the mean ± standard deviation (SD). The statistical analysis was performed using analysis of variance (ANOVA) followed by Dunnett's multiple range tests to determine levels of significance. AP-value < 0.05 was considered statistically significant.

## 3. Results

### 3.1. Effect of Chicory on Serum Uric Acid in Hyperuricaemic Rats with Renal Injury

**Figure 1:**
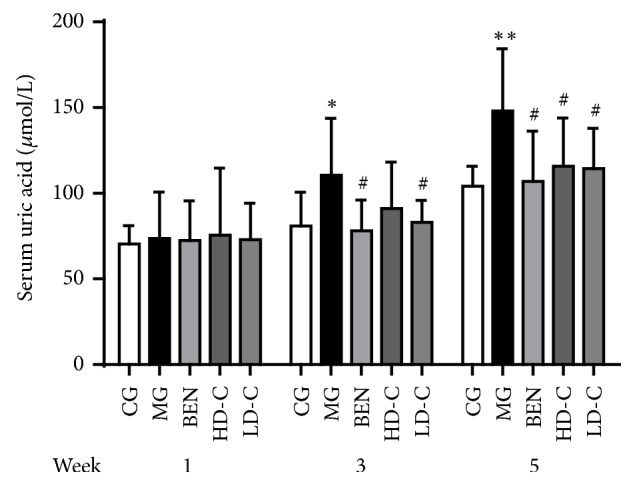
Effect of chicory on SUA in hyperuricaemic rats with renal injury.

As shown in Figure 1, the serum uric acid (SUA) levels of the model group (MG) were increased significantly at week 3 and week 5 (P<0.05, P<0.01) compared with those in the control group (CG). Compared with those in the MG, the SUA levels decreased significantly at week 3 and week 5 in the benzbromarone group (BEN) and the low dosage of chicory group (LD-C) (P<0.05) and decreased significantly at week 5 in the high dosage of chicory group (HD-C) (P<0.05) (Table S1).

### 3.2. Effect of Chicory on Serum Creatinine in Hyperuricaemic Rats with Renal Injury

**Figure 2:**
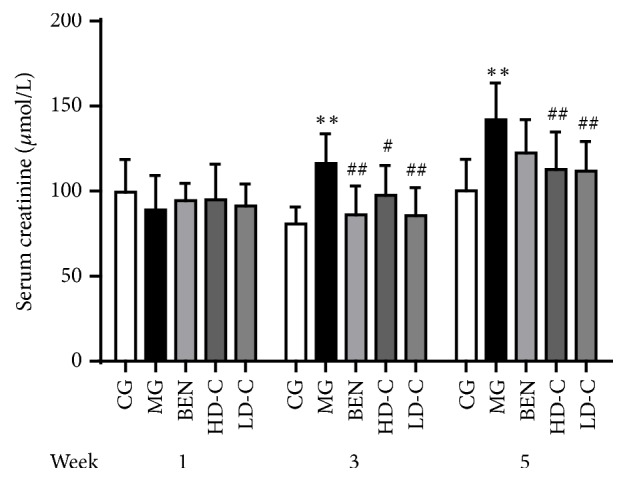
Effect of chicory on SCr in hyperuricaemic rats with renal injury.

As shown in Figure 2, the serum creatinine (SCr) levels of the MG were increased significantly at week 3 and week 5 (P<0.01) compared with those of the CG. Compared with those of the MG, the SCr level increased significantly at week 3 in the BEN (P<0.01) and decreased significantly in the HD-C and LD-C week 3 and week 5 (P<0.05, P<0.01) (Table S2).

### 3.3. Effect of Chicory on 24-h Urine Volume in Hyperuricaemic Rats with Renal Injury

**Figure 3:**
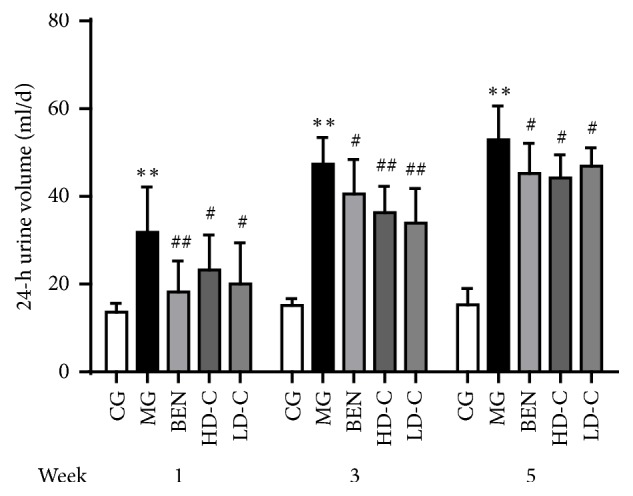
Effect of chicory on 24-h urine volume in hyperuricaemic rats with renal injury.

As shown in Figure 3, the 24-h urine volume was significantly higher at weeks 1, 3, and 5 (P<0.01) in the MG than in the CG. Compared with the volume in the MG, the volume at the same time point was significantly lower in the BEN and LD-C (P<0.05, P<0.01), and the volume in the HD-C was significantly lower at week 3 and week 5 (P<0.01, P<0.05) (Table S3).

### 3.4. Effect of Chicory on 24-h Urinary Uric Acid Excretion in Hyperuricaemic Rats with Renal Injury

**Figure 4:**
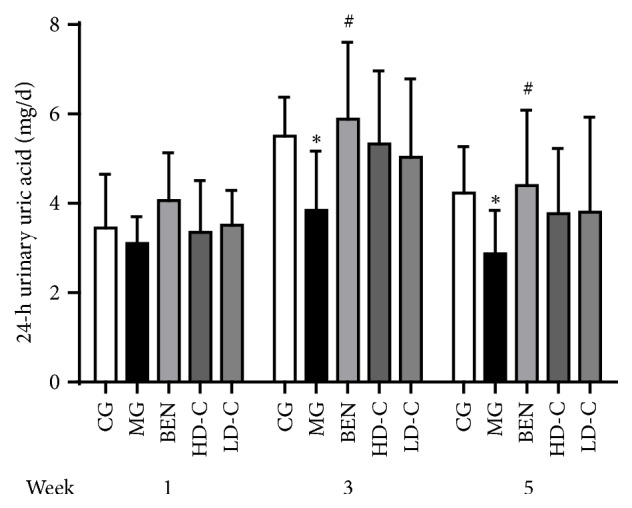
Effect of chicory on 24-h UUA excretion in hyperuricaemic rats with renal injury.

As shown in Figure 4, the 24-h urinary uric acid (UUA) excretion levels at week 3 and week 5 were significantly lower (P<0.05) in the MG than in the CG. Compared with those in the MG, the 24-h UUA excretion levels of the BEN were increased significantly at week 3 and week 5 (P<0.05), and the levels in the HD-C and LD-C showed an upward trend (Table S4).

### 3.5. Effect of Chicory on Creatinine Clearance in Hyperuricaemic Rats with Renal Injury

**Figure 5:**
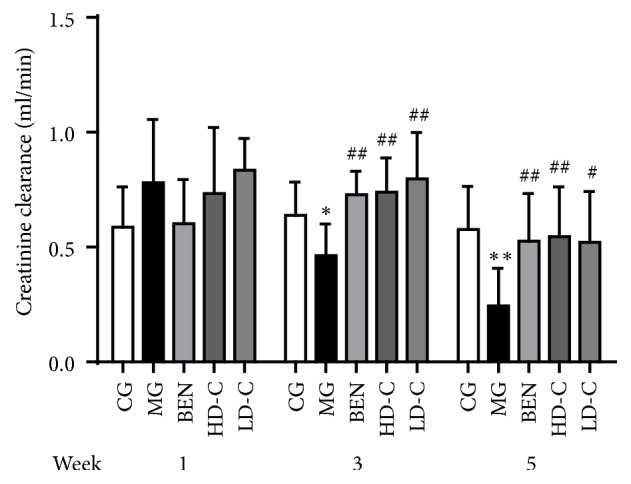
Effect of chicory on CrCl in hyperuricaemic rats with renal injury.

As shown in Figure 5, the creatinine clearance (CrCl) levels of the MG were decreased significantly at week 3 and week 5 (P<0.05, P<0.01) compared with those of the CG. The CrCl levels of the BEN, HD-C, and LD-C were significantly higher at the same time (P<0.05, P<0.01) than that in the MG (Table S5).

### 3.6. Effect of Chicory on 24-h Urinary Microalbumin Level in Hyperuricaemic Rats with Renal Injury

**Figure 6:**
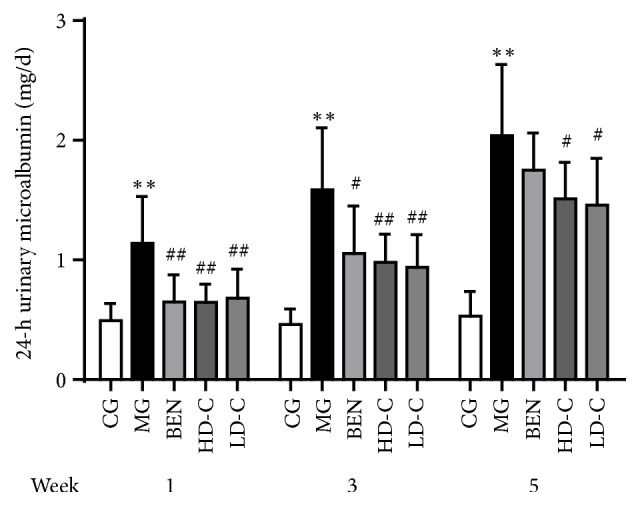
Effect of chicory on 24-h UMA in hyperuricaemic rats with renal injury.

As shown in Figure 6, the 24-h urinary microalbumin (UMA) level of the MG increased significantly at weeks 1, 3, and 5 (P<0.01) compared with that in the CG. Compared with the MG, all treatment groups showed a pronounced decrease (P<0.05, P<0.01), except for the BEN at week 5 (Table S6).

### 3.7. Effect of Chicory on Renal Histopathology in Hyperuricaemic Rats with Renal Injury

**Figure 7:**
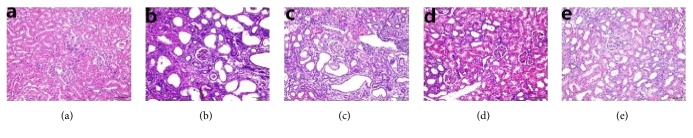
Glomerulus (HE, ×10 objective lens). (a) Control group; (b) hyperuricaemia with renal injury group; (c) group treated with benzbromarone; (d) group treated with the high dosage of chicory; (e) group treated with the low dosage of chicory.

**Figure 8:**
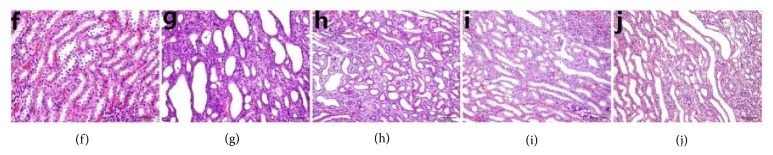
Renal tubule (HE, ×10 objective lens). (f) Control group; (g) hyperuricaemia with renal injury group; (h) group treated with benzbromarone; (i) group treated with the high dosage of chicory; (j) group treated with the low dosage of chicory.

As shown in Figures 7 and 8, haematoxylin and eosin (HE) stained kidney sections from the CG showed normal histological structures for the glomerulus, proximal convoluted tubules and distal convoluted tubules under light microscopy. There was tubular ectasia, and urate crystals were deposited in the tubules, with considerable inflammatory cell infiltration and focal fibrosis in the MG. The changes in tissue morphology, such as inflammatory cell infiltration, renal tubular ectasia and interstitial fibrosis, in the BEN, HD-C, and LD-C, were not evident as in the MG.

### 3.8. Effect of Chicory on Kidney GLUT9 mRNA Expression in Hyperuricaemic Rats with Renal Injury

**Figure 9:**
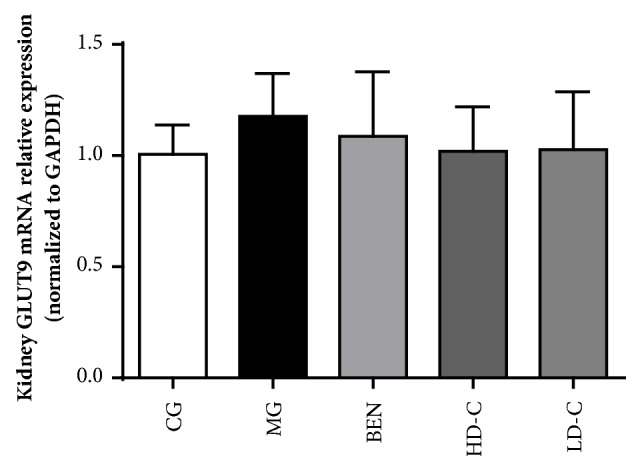
Effect of chicory on kidneys GLUT9 mRNA expression in hyperuricaemic rats with renal injury examined by RT-PCR.

Figure 9 shows that the GLUT9 (SLC2A9) mRNA expression levels were not different between any two groups (Table S7).

### 3.9. Effect of Chicory on Kidney GLUT9 Protein Expression in Hyperuricaemic Rats with Renal Injury

**Figure 10:**
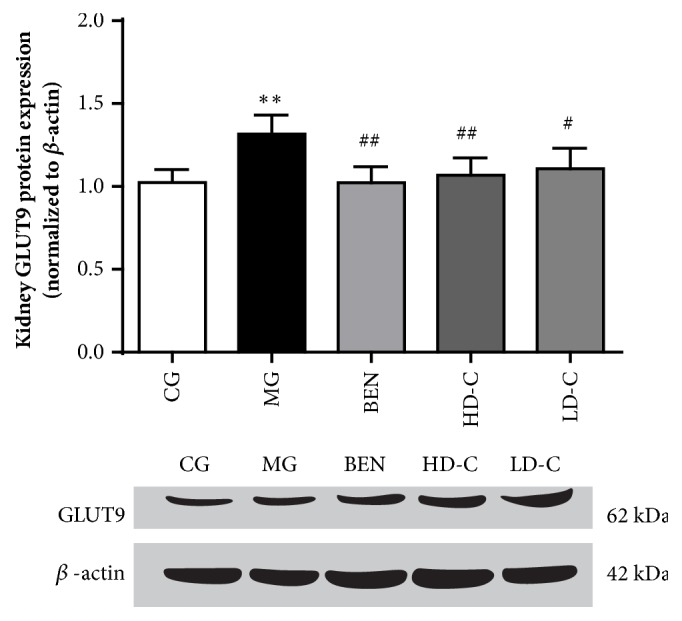
Effect of chicory on kidneys GLUT9 protein expression in hyperuricaemic rats with renal injury examined by western blotting.

As shown in Figure 10, the kidney GLUT9 protein expression in the MG increased significantly (P<0.01) compared with that in the CG, and its expression in the BEN, HD-C, and LD-C decreased significantly (P<0.01, P<0.05) compared with that in the MG (Table S8).

### 3.10. Effect of Chicory on the Proliferation of HKC Cells Determined Using the MTT Assay

As shown in Tables 2–4, UA concentrations greater than or equal to 400 *μ*mol/L significantly promoted the proliferation of HKC cells at 24 h and 48 h (P<0.05, P<0.01). Benzbromarone inhibited the proliferation of HKC cells, 200 *μ*mol/L significantly inhibited the proliferation (P<0.05) at 24 h, and 50 *μ*mol/L significantly inhibited the proliferation at 48 h (P<0.05). Chicory had no significant effect on HKC proliferation in the concentration range of 100-800 *μ*g/ml at 24 h.

### 3.11. Effect of Chicory on HKC Cells GLUT9 Protein Expression

**Figure 11:**
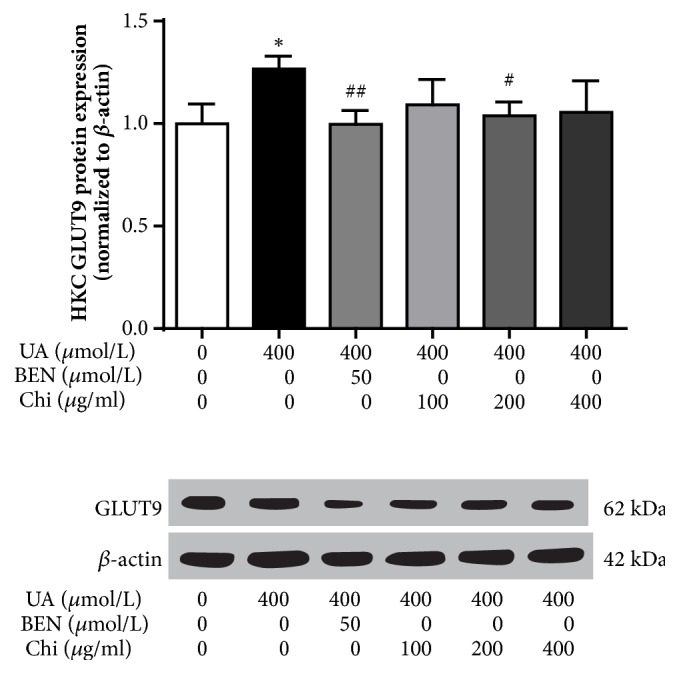
GLUT9 protein expression in HKC by western blotting.

As shown in Figure 11, the HKC cells GLUT9 protein expression increased significantly (P<0.05) when it stimulated with 400 *μ*mol/L UA for 24 h, and its expressions decreased significantly (P<0.01 or P<0.05) when it stimulated with 50 *μ*mol/L BEN or 200 *μ*g/ml Chi at the same time (Table S9).

### 3.12. Effect of Chicory on the Ability of Monolayer HKC Cells to Transport UA

**Figure 12:**
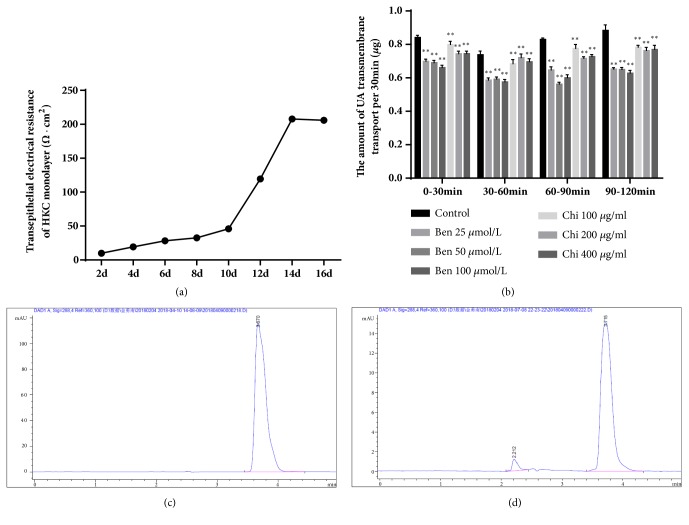
(a) The changes of TEER values in HKC cell monolayer; (b) the amount of UA transmembrane transport per 30 min; (c) the peak of UA from standard solution at 3.7 min; (d) the peak of UA from control group sample at 3.7 min.

As shown in Figure 12(a), the resistance of the monolayer of HKC cells on the Transwell polycarbonate membrane increased gradually, reached the peak (about 200 Ω•cm^2^) and then tended to be stable at 14-16 days. The concentration of UA standard substances in HBSS collected from the upper portions of the Transwell chamber at 30, 60, 90 and 120 min was determined by HPLC, and the amounts (*µ*g) of UA transferred from the lower chambers to the upper chambers were calculated every 30 min (Table S10).

The results showed in Figure 12(b) that transmembrane transport of UA to monolayer HKC cells was reduced after treatment with benzbromarone and chicory. When the concentrations of benzbromarone and chicory were in the range of 25-100 *μ*mol/L and 100-400 *μ*g/ml, respectively, the transmembrane transport of UA decreased with increasing drug concentration.

Figure 12(c) is the one peak of UA from standard solution. The peak appeared at 3.7 min, and the UA concentration is 16.810 *μ*g/ml.  Figure 12(d) is the peak of UA from control group sample at 3.7 min.

## 4. Discussion

In this study, the effect of chicory on serum uric acid (SUA), renal function and GLUT9 expression in the pathological state of hyperuricaemia rats with renal injury was investigated. Hyperuricaemia with renal injury was induced in rats by intragastric administration of yeast (15 g·kg^−1^·d^−1^) and adenine (80 mg·kg^−1^·d^−1^). The purine in yeast generates uric acid (UA) by the action of XOD and other enzymes in vivo. However, most mammals have uricase, which can catalyse UA oxidation to produce allantoin. Allantoin can dissolve in water and can be excreted from the kidney without being absorbed by renal tubules. Therefore, yeast is commonly used in combination with other agents, such as ethambutol and potassium oxonate, to induce hyperuricaemia in rats and mice [14–16]. For this purpose, we chose adenine, which is a nitrogenous heterocyclic purine compound. Adenine can increase phosphoribosyl pyrophosphate and glutamine in vivo to promote purine metabolism. On the other hand, adenine can become 2,8-dihydroxy adenine, which is extremely insoluble in water and crystallizes in the renal tubules, leading to chronic renal failure [17].

As expected, the model group (MG) displayed hyperuricaemia and renal function injury from week 3 to week 5. The levels of SUA and creatinine increased in the MG, and the 24-h urine volume also increased.

Creatinine clearance (CrCl) is an indicator that can reflect renal function more accurately and sensitively than serum creatinine [18], and the endogenous creatinine clearance rate is a sensitive index for evaluating impaired glomerular filtration. The results showed that, in the MG, CrCl decreased to 72.7% and 42.3% at week 3 and week 5, respectively. At the same time, for treatment with benzbromarone, high dosage and low dosage of chicory, the CrCl reached 114.3%, 116.0%, and 124.9% at week 3 and 91.2%, 94.6%, and 90.1% at week 5, respectively. This finding indicated that benzbromarone and chicory can alleviate the kidney damage caused by yeast and adenine in rats.

As shown with haematoxylin and eosin (HE) staining, the numbers of glomeruli in the high dosage and low dosage of chicory groups were greater than those in the model and benzbromarone groups, and the renal tubular expansion was less than that in the model and benzbromarone groups. These changes support the protective effect of chicory on renal function.

Excretion of UA is mainly determined by the balance between renal reabsorption and secretion, and the role of various urate transporters has been debated [8]. Usually, GLUT9, URAT1, OAT1, and OAT3 are the main transporters regulating renal urate handling, while ABCG2 appears to regulate intestinal transport [19]. For example, Vibha Bhatnagar et al. pointed out that Chronic Renal Insufficiency Cohort data showed that GLUT9 played a much less significant role than ABCG2 in this subset of patients with CKD [20]. Similarly, Hirofumi Yano et al. observed that serum UA level did not increase despite the decrease in renal UA excretion in the 5/6 nephrectomy rats model [21]. These studies in patients of CKD and rodent animals showed that renal urate transport was affected during CKD, and ABCG2 may play a compensatory role and compensate for the extra-renal excretion.

In addition, GLUT9 (SLC2A9) is known to transport glucose or fructose until Doblado and Moley reported that GLUT9 may transport not only fructose but also urate [22–24], and this study is focused on GLUT9 and renal urate excretion. At present, GLUT9 is thought to be a high capacity and low affinity urate transporter [25–27]. GLUT9 is located on the basolateral membrane of renal tubular epithelial cells. Hamajima et al. [28], through the analysis of candidate genes from 5024 healthy Japanese individuals, found a significant correlation between SLC2A9 rs11722228 and the level of SUA. Research from Wang M and Yang H showed that SUA was reduced in hyperuricaemic mice and that renal UA excretion was promoted in hyperuricaemic rats when the expression of GLUT9 was inhibited [29, 30].

As shown in the qPCR and western blot results, there was no significant change in GLUT9 mRNA expression in each group of kidney tissues. However, GLUT9 protein expression in the kidney increased markedly to 128.3% in hyperuricaemic rats with renal injury, and the expression levels decreased to 77.8%, 81.2%, and 84.3%, respectively, when the animals were treated with benzbromarone, high dosage chicory and low dosage chicory. This result indicated that chicory could inhibit the expression of GLUT9 protein in the kidneys of hyperuricaemic rats with renal injury. As a result, the UA reabsorption in the renal tubules is reduced, and the UA excretion by renal tubules is increased. Therefore, chicory plays a role in reducing SUA levels.

Cell experiments were performed in vitro to further verify that the effect of chicory on reducing UA is related to the regulation of GLUT9 protein expression. Benzbromarone was used as an inhibitor to inhibit GLUT9 in HKC cells, and the effects of chicory on protein expression and the ability of monolayer HKC cells to transport UA were evaluated to verify that chicory lowers SUA by regulating GLUT9 protein expression.

HKC cells are a group of cells that are mostly composed of normal human proximal tubular epithelial cells. HKC cells are widely used in pharmacological and toxicological experiments [31, 32]. GLUT9 is mainly distributed on the tubular apical membrane of the renal tubule. Protein was extracted from HKC cells and incubated with GLUT9 (SLC2A9) primary antibody, and its protein band was located at 62 kDa. After 24 h of UA stimulation, the expression increased 26.7%, benzbromarone decreased the expression to 21.3%, and chicory (200 *μ*g/ml) decreased the expression to 18.1%. The western blot results of the HKC cells were consistent with the results of the kidney tissue.

The effect of chicory on the expression of GLUT9 protein has been clarified. To further observe the effect of chicory on GLUT9 function, a Transwell assay was used for UA transport experiments. Literature reports indicate that Tranilast can inhibit urate transport through URAT1 and GLUT9 in a reversible, noncompetitive manner [33] and that benzbromarone can also inhibit GLUT9 and URAT1 [34]. Therefore, the decrease in UA transport in the Transwell assay is not only caused by changes in GLUT9 expression levels but also may be affected by changes in the expression levels of other uric acid transporters, such as URAT1. However, the GLUT9 protein band was incubated with a specific primary antibody against GLUT9 (SLC2A9), and chicory reduced the GLUT9 protein expression on HKC cells induced by UA stimulation. Therefore, combining the two results indicate that chicory can inhibit the expression of GLUT9 protein in renal tubules and that this inhibition affects the ability of GLUT9 to transport UA.

Finally, knockout mice are used in the research of urate transporters at present. It is reported that OAT1 and OAT3 knockout mice without obvious histological or anatomic abnormalities, and they are similar to their wild-type counterparts with respect to metabolic parameters. Thus the knockout mice could use assessment of renal transport of urate and other organic anions [8]. Therefore, our further research may be to select gene knockout rats or mice to verify the conclusions of this study.

## 5. Conclusion

The study indicated that chicory lowered serum uric acid levels and alleviated renal function in hyperuricaemic rats with renal injury. In vitro and in vivo biochemical experiments showed that chicory acts on the renal uric acid transporter GLUT9 and down regulates its protein expression level as a mechanism for reducing uric acid. The experiment showed that the chicory had a better effect than benzbromarone in delaying the development of renal function damage caused by adenine in model animals, indicating the possibility of using chicory as an alternative to alleviating renal damage in hyperuricaemia.

## Figures and Tables

**Table 1:** Primers used for the quantitative RT-PCR gene expression studies.

Gene	Forward primer	Reverse primer
GLUT9	5′-TGCA TTGG CGTG TTTT CTGG-3′	5′-GTTT GGAA GGCT TTCG TGGC-3′
GAPDH	5′-GGTG GACC TCAT GGCC TACA-3′	5′-ATTG TGAG GGAG ATCC TCAG TGT-3′

**Table 2:** Comparison of HKC cells proliferation ability at 24 h and 48 h with various concentrations of UA ($\overset{-}{x}$±s, n=6).

UA (*μ*mol/L)	24 h	48 h
0 (control group)	0.2959 ± 0.0267	0.4022 ± 0.0333
100	0.3121 ± 0.0242	0.4223 ± 0.0203
200	0.3257 ± 0.0367	0.4406 ± 0.0345
400	0.3389 ± 0.0245*∗*	0.4522 ± 0.0273*∗*
600	0.3528 ± 0.0394*∗*	0.4851 ± 0.0375*∗∗*
800	0.3742 ± 0.0263*∗∗*	0.4963 ± 0.0294*∗∗*

*Notes.∗* P<0.05, *∗∗* P<0.01 vs CG.

**Table 3:** Comparison of HKC cells proliferation ability at 24 h and 48 h with various concentrations of benzbromarone ($\overset{-}{x}$±s, n=6).

Benzbromarone (*μ*mol/L)	24h	48h
0 (control group)	0.3065 ± 0.0216	0.4209 ± 0.0267
12.5	0.2992 ± 0.0297	0.4092 ± 0.0360
25	0.3043 ± 0.0305	0.3768 ± 0.0364*∗*
50	0.2790 ± 0.0237	0.3370 ± 0.0381*∗∗*
100	0.2756 ± 0.0347	0.3377 ± 0.0352*∗∗*
200	0.2551 ± 0.0384*∗*	0.3177 ± 0.0426*∗∗*

*Notes.∗* P<0.05, *∗∗* P<0.01 vs CG.

**Table 4:** Comparison of HKC cells proliferation ability at 24 h and 48 h with various concentrations of chicory ($\overset{-}{x}$±s, n=6).

Chicory (*μ*g/ml)	24 h	48 h
0 (control group)	0.2859 ± 0.0230	0.4097 ± 0.0433
100	0.2858 ± 0.0188	0.3783 ± 0.0464
200	0.2657 ± 0.0240	0.3724 ± 0.0501
400	0.2644 ± 0.0354	0.3611 ± 0.0459
600	0.2726 ± 0.0221	0.3408 ± 0.0317*∗*
800	0.2602 ± 0.0475	0.3421 ± 0.0483*∗*

*Notes.∗* P<0.05.

## Data Availability

The data used to support the findings of this study are included within the article.

## Supplementary Materials

Supplementary Materials Table S1: The effect of chicory on SUA in hyperuricaemic rats with renal injury. Table S2: The effect of chicory on SCr in hyperuricaemic rats with renal injury. Table S3: The effect of chicory on 24-h urine volume in hyperuricaemic rats with renal injury. Table S4: The effect of chicory on 24-h UUA excretion in hyperuricaemic rats with renal injury. Table S5: The effect of chicory on CrCl in hyperuricaemic rats with renal injury. Table S6: The effect of chicory on 24-h UMA in hyperuricaemic rats with renal injury. Table S7: The effect of chicory on kidneys GLUT9 mRNA expression in hyperuricaemic rats with renal injury. Table S8: The effect of chicory on kidneys GLUT9 protein expression in hyperuricaemic rats with renal injury. Table S9: The effect of chicory on GLUT9 protein expression in HKC cells. Table S10: The changes of TEER values in HKC cell monolayer (Supplementary Materials).
