# Corrigendum to “Effects of Chicory on Serum Uric Acid, Renal Function, and GLUT9 Expression in Hyperuricaemic Rats with Renal Injury and In Vitro Verification with Cells”

**DOI:** 10.1155/2022/9801719

**Published:** 2022-04-13

**Authors:** Yongnan Jin, Zhijian Lin, Bing Zhang, Yun-Fei Bai

**Affiliations:** ^1^Department of Clinical Chinese Pharmacy, School of Chinese Pharmacy, Beijing University of Chinese Medicine, Beijing 100029, China; ^2^Department of Integrated TCM and Western Medicine, Yanbian University Hospital, Yanji 133000, China

In the article titled “Effects of Chicory on Serum Uric Acid, Renal Function, and GLUT9 Expression in Hyperuricaemic Rats with Renal Injury and In Vitro Verification with Cells” [1], concerns about the western blots presented in Figures 1 and 2 were raised on PubPeer [2]. Specifically, the backgrounds of the blots appear to be unusually clean. The authors have explained that the error occurred when changing the format of the image files during the production of the article, and the journal has confirmed that this issue was not present in the image files provided during the peer review process. These original images are as follows.

## Figures and Tables

**Figure 1:**
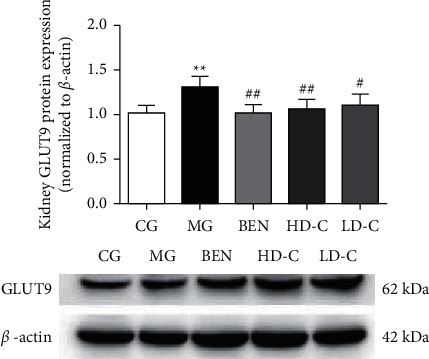
Effect of chicory on kidneys GLUT9 protein expression in hyperuricaemic rats with renal injury examined by western blotting.

**Figure 2:**
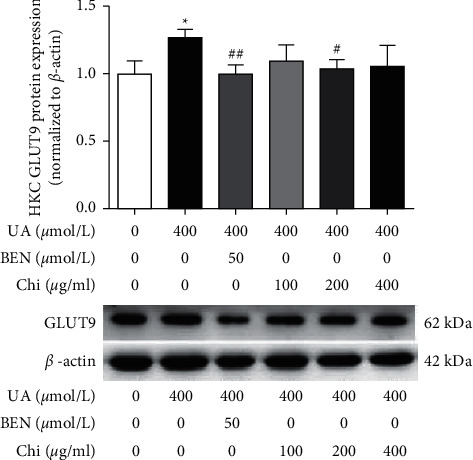
GLUT9 protein expression in HKC by western blotting.
