# Supplementary material for: Effects of Chicory on Serum Uric Acid, Renal Function, and GLUT9 Expression in Hyperuricaemic Rats with Renal Injury and In Vitro Verification with Cells
Source: Evid Based Complement Alternat Med. 2018 Dec 2;2018:1764212. doi: 10.1155/2018/1764212 (PMC6304617; doi:10.1155/2018/1764212)
Supplement: Supplementary Materials — Table S1: The effect of chicory on SUA in hyperuricaemic rats with renal injury. Table S2: The effect of chicory on SCr in hyperuricaemic rats with renal injury. Table S3: The effect of chicory on 24-h urine volume in hyperuricaemic rats with renal injury. Table S4: The effect of chicory on 24-h UUA excretion in hyperuricaemic rats with renal injury. Table S5: The effect of chicory on CrCl in hyperuricaemic rats with renal injury. Table S6: The effect of chicory on 24-h UMA in hyperuricaemic rats with renal injury. Table S7: The effect of chicory on kidneys GLUT9 mRNA expression in hyperuricaemic rats with renal injury. Table S8: The effect of chicory on kidneys GLUT9 protein expression in hyperuricaemic rats with renal injury. Table S9: The effect of chicory on GLUT9 protein expression in HKC cells. Table S10: The changes of TEER values in HKC cell monolayer (Supplementary Materials). [file 1764212.f1.docx]

Supplementary materials

（1）Serum uric acid (SUA)

Table S1: Effect of chicory on SUA in hyperuricaemic rats with renal injury (μmol/L, x̄±s, n=12).

| Groups | Week 1 | Week 3 | Week 5 |
| --- | --- | --- | --- |
| CG | 70.39±10.63 | 80.92±19.62 | 104.12±11.70 |
| MG | 73.51±27.24 | 110.58±33.17* | 148.07±36.34** |
| BEN | 72.37±23.26 | 78.13±17.96# | 106.96±29.23# |
| HD-C | 75.65±39.17 | 91.14±27.07 | 115.68±28.22# |
| LD-C | 72.94±21.25 | 83.03±12.84# | 114.40±23.54# |

Notes: * P<0.05, ** P<0.01 vs CG; # P<0.05 vs MG.

（2）Serum creatinine (SCr)

Table S2: Effect of chicory on SCr in hyperuricaemic rats with renal injury (μmol/L, x̄±s, n=12).

| Groups | Week 1 | Week 3 | Week 5 |
| --- | --- | --- | --- |
| CG | 99.35±19.18 | 80.81±9.93 | 100.22±18.49 |
| MG | 88.87±20.38 | 116.33±17.42** | 141.87±21.66** |
| BEN | 94.46±10.08 | 86.07±17.00## | 122.34±19.66 |
| HD-C | 94.93±21.06 | 97.55±17.48# | 112.83±21.88## |
| LD-C | 91.20±13.03 | 85.59±16.50## | 111.76±17.56## |

Notes: ** P<0.01 vs CG; # P<0.05, ## P<0.01 vs MG.

（3）24-h urine volume

Table S3: Effect of chicory on 24-h urine volume in hyperuricaemic rats with renal injury (ml/d, x̄±s, n=10).

| Groups | Week 1 | Week 3 | Week 5 |
| --- | --- | --- | --- |
| CG | 13.65±1.96 | 15.15±1.58 | 15.30±3.76 |
| MG | 31.85±10.33** | 47.35±6.05** | 52.89±7.76** |
| BEN | 18.20±7.09## | 40.60±7.84# | 45.22±6.85# |
| HD-C | 22.70±7.11# | 36.30±5.98## | 44.20±5.28# |
| LD-C | 20.05±9.38# | 33.90±7.90## | 46.90±4.20# |

Notes: ** P<0.01 vs CG;# P<0.05, ## P<0.01 vs MG.

（4）24-h UUA excretion

Table S4: Effect of chicory on 24-h UUA excretion in hyperuricaemic rats with renal injury (mg/d, x̄±s, n=10).

| Groups | Week 1 | Week 3 | Week 5 |
| --- | --- | --- | --- |
| CG | 3.45±1.20 | 5.50±0.87 | 4.23±1.04 |
| MG | 3.10±0.60 | 3.84±1.33* | 2.87±0.97* |
| BEN | 4.06±1.07 | 5.88±1.72# | 4.40±1.69# |
| HD-C | 3.35±1.16 | 5.33±1.63 | 3.77±1.46 |
| LD-C | 3.51±0.78 | 5.03±1.76 | 3.80±2.13 |

Notes: * Pvs CG; # P<0.05 vs MG.

（5）Creatinine clearance (CrCl)

Table S5: Effect of chicory on CrCl in hyperuricaemic rats with renal injury (ml/min, x̄±s, n=10).

| Groups | Week 1 | Week 3 | Week 5 |
| --- | --- | --- | --- |
| CG | 0.586±0.176 | 0.638±0.146 | 0.577±0.188 |
| MG | 0.781±0.275 | 0.464±0.137* | 0.244±0.164** |
| BEN | 0.602±0.192 | 0.729±0.102## | 0.526±0.208## |
| HD-C | 0.734±0.286 | 0.740±0.148## | 0.546±0.217## |
| LD-C | 0.835±0.139 | 0.797±0.203## | 0.520±0.222# |

Notes: * P<0.05, ** P<0.01 vs CG; # P<0.05, ## P<0.01 vs MG.

（6）24-h Urinary microalbumin (UMA)

Table S6: Effect of chicory on 24-h UMA in hyperuricaemic rats with renal injury (mg/d, x̄±s, n=10).

| Groups | Week 1 | Week 3 | Week 5 |
| --- | --- | --- | --- |
| CG | 0.494±0.142 | 0.460±0.131 | 0.531±0.205 |
| MG | 1.139±0.392** | 1.587±0.517** | 2.040±0.595** |
| BEN | 0.648±0.228## | 1.053±0.397# | 1.751±0.309 |
| HD-C | 0.647±0.151## | 0.981±0.235## | 1.512±0.305# |
| LD-C | 0.682±0.242## | 0.938±0.273## | 1.460±0.390# |

Notes: ** P<0.01 vs CG; # P<0.05, ## P<0.01 vs MG.

（7）Kidney GLUT9 mRNA expression

Table S7: Effect of chicory on kidneys GLUT9 mRNA expression in hyperuricaemic rats with renal injury (mg/d, x̄±s, n=10).

| Groups | 2^-ΔΔCt^ |
| --- | --- |
| CG | 1.006±0.133 |
| MG | 1.176±0.193 |
| BEN | 1.087±0.291 |
| HD-C | 1.020±0.200 |
| LD-C | 1.027±0.261 |

（8）Kidney GLUT9 protein expression

Table S8: Effect of chicory on kidneys GLUT9 protein expression in hyperuricaemic rats with renal injury (x̄±s, n=5).

| Groups | GLUT9/β-actin |
| --- | --- |
| CG | 1.029±0.078 |
| MG | 1.315±0.116** |
| BEN | 1.023±0.097## |
| HD-C | 1.068±0.104## |
| LD-C | 1.108±0.123# |

Notes: ** P<0.01 vs CG; # P<0.05, ## P<0.01 vs MG.

（9）GLUT9 protein expression in HKC cells

Table S9: effect of chicory on GLUT9 protein expression in HKC cells (x̄±s, n=3).

| Groups | GLUT9/β-actin |
| --- | --- |
| CG | 1.000±0.096 |
| 400 μmol/L UA | 1.267±0.062* |
| 400 μmol/L UA+50 μmol/L BEN | 0.996±0.068## |
| 400 μmol/L UA+100 μg/ml Chi | 1.092±0.123 |
| 400 μmol/L UA+200 μg/ml Chi | 1.038±0.067# |
| 400 μmol/L UA+400 μg/ml Chi | 1.056±0.153 |

Notes: * P<0.05 vs CG; # P<0.05, ## P<0.01 vs. 400 μmol/L UA

（10）TEER values in HKC cell monolayer

Table S10: The changes of TEER values in HKC cell monolayer (Ω•cm^2^, x̄±s, n=6)

| time | TEER |
| --- | --- |
| 2d | 9.79±4.88 |
| 4d | 19.21±3.20 |
| 6d | 28.25±6.63 |
| 8d | 32.58±4.71 |
| 10d | 45.95±6.73 |
| 12d | 119.40±17.12 |
| 14d | 207.73±20.21 |
| 16d | 205.85±20.63 |
